# Regenerative Approaches in Huntington’s Disease: From Mechanistic Insights to Therapeutic Protocols

**DOI:** 10.3389/fnins.2018.00800

**Published:** 2018-11-02

**Authors:** Jenny Sassone, Elsa Papadimitriou, Dimitra Thomaidou

**Affiliations:** ^1^Vita-Salute University and San Raffaele Scientific Institute, Milan, Italy; ^2^Department of Neurobiology, Hellenic Pasteur Institute, Athens, Greece

**Keywords:** Huntington’s disease, iPCs, direct reprogramming, neuroprotection, *in vivo* reprogramming, miRNAs

## Abstract

Huntington’s Disease (HD) is a neurodegenerative disorder caused by a CAG expansion in the exon-1 of the IT15 gene encoding the protein Huntingtin. Expression of mutated Huntingtin in humans leads to dysfunction and ultimately degeneration of selected neuronal populations of the striatum and cerebral cortex. Current available HD therapy relies on drugs to treat chorea and control psychiatric symptoms, however, no therapy has been proven to slow down disease progression or prevent disease onset. Thus, although 24 years have passed since HD gene identification, HD remains a relentless progressive disease characterized by cognitive dysfunction and motor disability that leads to death of the majority of patients, on average 10–20 years after its onset. Up to now several molecular pathways have been implicated in the process of neurodegeneration involved in HD and have provided potential therapeutic targets. Based on these data, approaches currently under investigation for HD therapy aim on the one hand at getting insight into the mechanisms of disease progression in a human-based context and on the other hand at silencing mHTT expression by using antisense oligonucleotides. An innovative and still poorly investigated approach is to identify new factors that increase neurogenesis and/or induce reprogramming of endogenous neuroblasts and parenchymal astrocytes to generate new healthy neurons to replace lost ones and/or enforce neuroprotection of pre-existent striatal and cortical neurons. Here, we review studies that use human disease-in-a-dish models to recapitulate HD pathogenesis or are focused on promoting *in vivo* neurogenesis of endogenous striatal neuroblasts and direct neuronal reprogramming of parenchymal astrocytes, which combined with neuroprotective protocols bear the potential to re-establish brain homeostasis lost in HD.

## Introduction

Huntington’s Disease (HD) is an autosomal-dominant neurodegenerative disorder with prevalence of ∼7–11 per 100,000 in the caucasian population (Spinney, 2010). It is caused by abnormal expansion of a trinucleotide CAG repeat in exon 1 of the HTT gene (MacDonald et al., 1993) and is characterized by severe motor, cognitive and psychiatric symptoms. HD neuropathology is characterized by preferential degeneration of GABAergic medium spiny neurons (MSNs) of the striatum and, in a lesser extent, of pyramidal projection neurons in cortical layers V, VI, and III, innervating the striatum (Cudkowicz and Kowall, 1990). Neurodegeneration in HD is preceded by a long period of neuronal dysfunction, associated with transcriptional and epigenetic changes resulting in progressive loss of striatal identity (Seredenina and Luthi-Carter, 2012; Langfelder et al., 2016). Neurodegeneration in HD may also be accompanied by decreased striatal neurogenesis, a fact that may also account for part of HD symptomatology (Ernst et al., 2014). As of now, molecular mechanisms underlying HD pathogenesis remain elusive, and no therapeutic treatments are currently available beyond clinical symptomatic management.

**FIGURE 1:**
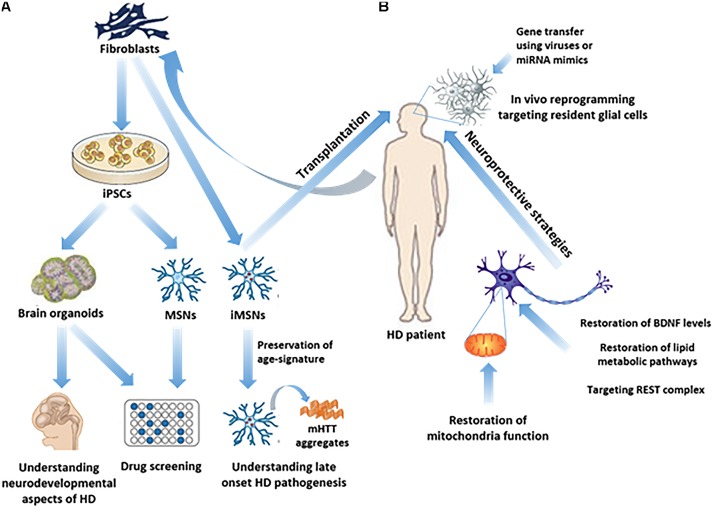
Study of HD pathogenesis and possible therapeutic approaches in humans. **(A)** iPSC technology has made it possible to generate *in vitro* brain organoids or subtype specific neuronal populations related to HD, such as MSNs, from HD patients’ fibroblasts, thus enabling the study of the neurodevelopmental aspects of HD, as well as drug screening. Direct reprogramming of patients’ fibroblasts to induced-MSNs (iMSNs) facilitated the study of late onset HD pathogenesis, since iMSNs retain age-related signatures. **(B)** *In vivo* regenerative approaches as possible therapeutic strategies, including transplantation of iMSNs or direct reprogramming of resident glial cells into induced neurons in the striatum, remain to be further explored. On the other hand, neuroprotective approaches have been more extensively explored aiming either at targeting REST complex activity, or at the restoration of BDNF levels, lipid metabolic pathways or mitochondrial function.

An effective therapy in HD may use a combined approach of cell *trans*-differentiation and neuroprotection. The following chapters review the main identified molecular pathways and potential therapeutic targets which can lead to the development of cell reprogramming and neuroprotective protocols (Figure 1).

## Cell Replacement Approaches in Animal Models of HD

A variety of rodent models have been created to recapitulate neuropathological features and symptoms of either juvenile, early adult, or adult human HD (Mangiarini et al., 1996; Slow et al., 2003) and to develop cell therapy protocols using renewable cell sources, including fetal neural stem cells (NSCs), embryonic stem cells (ESCs), induced pluripotent stem cells (iPSCs) and induced neural stem cells (iNSCs) for brain repair in HD (for review see (Tartaglione et al., 2017). The majority of recent transplantation studies were performed using the Quinolinic Acid (QA) excitotoxic lesion model, as it induces a selective loss of striatal MSNs with a relative preservation of interneurons, largely resembling the neuropathological features of human HD (Beal et al., 1991). In these studies human progenitor cells, either hESCs or hiPSCs were, prior to their transplantation, *in vitro* differentiated to striatal progenitors or immature MSNs, either through directed differentiation protocols modulating the levels of extrinsic developmental signals, such as BMP/TGFβ (Carri et al., 2013), Sonic Hedgehog (SHH) and Activin A (Arber et al., 2015) or by forced expression of transcription factors (TFs) involved in MSNs differentiation, such as GSX2 and EBF1 (Faedo et al., 2017). In these studies, transplantation of the enriched populations of striatal progenitors resulted in their functional integration into the lesioned striatum, a subpopulation of which differentiated to DARPP-32^+^ MSNs (Arber et al., 2015), extended fibers over a long distance (Faedo et al., 2017), projected to the substantia nigra and received GABAergic and glutamatergic inputs, leading to restoration of apomorphine-induced rotational behavior (Ma et al., 2012). In a very recent study, a hydrogel scaffold has been used for the more effective, rapid and scalable directed differentiation of human iPSCs to striatal progenitors in three-dimensional (3D) organoid-like structures (Adil et al., 2018). 3D-derived striatal progenitors grafted into R6/2 HD mice (Mangiarini et al., 1996), developed an MSN-like phenotype and formed synaptic connections with host cells, resulting in improvement of mice motor coordination (Adil et al., 2018). Although the use of human cells – in particular healthy hESCs that partially overcomes the problem of dealing with a diseased system – bears potential for cell replacement, studies are still in a preliminary stage and more rigorous testing of human cell directed differentiation on 2D and 3D culture systems and transplantation in various HD animal models is needed to assess both circuit reconstruction and behavioral recovery in HD.

## *In Vitro* Differentiation for HD Modeling

Whilst HD rodent models have undoubtedly yielded much useful data, the nature of these systems makes insights gained from such a stand-alone model limited when it comes to translation in human patients. On the other hand, cell transplantation has only partially restored lost function in pre-clinical models and clinical trials. To this end the discovery and advancement of iPSCs technology has allowed for a more thorough study of human HD on a cellular and developmental level. The first iPSC lines were generated from HD patients (Park et al., 2008) and since then, many iPSC-based human HD cell models with different CAG repeat lengths have been generated, among which, the ones generated by the HD iPSC Consortium are the best characterized (Jeon et al., 2012; The HD iPSC Consortium, 2012). HD iPSCs and the neural cell types derived from them recapitulate some disease phenotypes found in both human patients and animal models, such as altered cell growth (Jeon et al., 2012), cell adhesion, survival, electrophysiological properties, metabolism (The HD iPSC Consortium, 2012), protein clearance (proteasomal, autophagic), oxidative stress/antioxidant response (Szlachcic et al., 2015) and mitochondrial fragmentation (Guo et al., 2013). Interestingly, gene expression studies have revealed that neurons derived from HD iPSCs exhibit deregulated signaling pathways directly related to development and neurogenesis. Conforti et al., 2018 showed that early telencephalic induction and late neural identity are affected in cortical and striatal populations obtained from HD iPSC lines. It was also reported for the first time using cortical organoids that large CAG expansion causes complete failure of the neuro-ectodermal acquisition, while cells carrying shorter CAG repeats show gross abnormalities in neural rosette formation as well as disrupted cytoarchitecture (Conforti et al., 2018). Interestingly, gene-expression analysis revealed that control organoids overlapped with differentiated human fetal cortical areas, while HD organoids correlated with the immature ventricular / subventricular zone (Conforti et al., 2018). Along the same lines, recent data using isogenic human embryonic stem cell (hESC) lines, suggest that HD is caused by chromosomal instability and begins far earlier than expected as a dominant-negative loss-of function, rather than through the broadly accepted gain-of toxic function mechanism (Ruzo et al., 2018). This evidence supports the hypothesis that an early neurodevelopmental defect exists in HD and could contribute to the later adult neurodegenerative phenotype (Lim et al., 2017; Wiatr et al., 2018).

Considering this pathological phenotype, PSCs, including iPSCs and ESCs, have been used to screen for HD therapies. A screen in wild type human ESCs-derived neural stem cells (NSCs) for chemical inhibitors of the transcriptional repressor REST resulted in the identification of one potent compound, named X5050, able to increase the expression of neuronal genes targeted by REST in wild type neural cells (Charbord et al., 2013). Acute intraventricular delivery of this small molecule increased the expression of the key neurotrophic factor BDNF, being depleted during disease progression (Zuccato et al., 2001), as well as, several other REST-regulated genes in the prefrontal cortex of mice with QA-induced striatal lesions (Charbord et al., 2013), highlighting its potential therapeutic value in HD. In another study led by the HD iPSC Consortium, RNA-seq analysis in iPSC-derived neural cultures revealed consistent deficits related to neurodevelopmental gene networks and led to the identification of a small molecule, isoxazole-9 (Isx-9), that targeted several of these dysregulated networks and successfully normalized CAG repeat-associated phenotypes in both juvenile- and adult-onset HD iPSC-derived neural cultures, as well as cognition and synaptic pathology in R6/2 HD mice (Lim et al., 2017).

Hence, these results highlight that ESC/iPSC are promising cellular models for the investigation of the molecular defects underlying HD pathogenesis and the screening of compounds for HD therapies. Important caveat remains the need for optimization of human iPSC models, including reprogramming and differentiation protocols, for the purposes of consistent observation. Importantly, given the fact that human iPSCs become rejuvenated by erasing epigenetic aging signatures (Mertens et al., 2015), they seem more appropriate for studying early onset diseases and this is probably the reason that the majority of results obtained from studies using iPSCs is derived from individuals with juvenile onset HD (>60 CAGs) rather than with adult onset HD (39–60 CAGs) (Park et al., 2008; Guo et al., 2013; Szlachcic et al., 2015). The contribution of epigenetic aging signature in the appearance of HD pathology is also supported by the finding that human iPSC-derived lines with expansion lower than 60 CAG repeats, corresponding to late onset HD pathology, don’t exhibit major observable pathological phenotypes (Mattis and Svendsen, 2018).

### Direct Reprogramming in HD: Preservation of Age-Related Signatures

Accordingly it has been recently established that modeling an adult-onset disorder might require the maintenance of aging signatures. Along this line, direct reprogramming approaches that result in the production of neuronal types that retain donor age-dependent aging signatures, such as age-specific transcriptional profiles, nucleo-cytoplasmic compartmentalization and an aged DNA methylation epigenetic clock (Mertens et al., 2015), seem to be appropriate human models for the study of HD, which is primarily an adult-onset disease. Victor et al., 2014 reported for the first time the direct reprogramming of human fibroblasts into induced-MSNs (i-MSNS), by force-expressing neurogenic miRNAs miR-9/9^∗^-miR-124 together with the TFs CTIP2, DLX1/2, and MYT1L. Interestingly, when the i-MSNs were transplanted into the mouse striatum they survived for more than 6 months and projected to their correct targets (Victor et al., 2014). Recently the same group expanded this protocol for the *in vitro* generation of i-MSNs from healthy and HD-patients’ fibroblasts (Victor et al., 2018). Remarkably, HD neurons generated in this manner displayed inclusion-body formation, mitochondrial and metabolic dysfunction and cell death, mirroring the defects occurring in the striatum of HD patients. By contrast, heMSNs derived by direct conversion of human HD embryonic fibroblasts (HEFs), produced by differentiation of HD-iPSCs, exerted a milder HD phenotype with lower mHTT aggregation, supporting the notion that the age-related decline in protein homeostasis could contribute to HD pathology. Taken together the findings of this study presented for the first time a patient-based platform of MSNs for the study of the age-related mechanisms of late onset HD pathogenesis and proved that in a human context cellular age is an essential component underlying the manifestation of HD phenotypes and that age-associated reduction in protein homeostasis levels is primarily responsible for mHTT aggregation in HD i-MSNs.

## *In Vivo* Reprogramming for HD Therapy

A novel strategy for cell replacement therapy for HD is the concept of *in vivo* cell reprogramming in the striatum, which is the area mostly affected. Increasing evidence indicates that astrocytes within the striatal parenchyma can undergo endogenous *trans*-differentiation into neuroblasts after stroke or QA-mediated excitotoxic lesion that induces selective loss of striatal MSNs (Magnusson et al., 2014; Nato et al., 2015), revealing the intrinsic existence of a latent neurogenic capacity in the adult striatum. These cells were found to express markers of immature newborn neurons like ASCL1 or DCX, while some of them developed into mature NeuN^+^ neurons, several of which expressed MSN TFs (Luzzati et al., 2011) and the MSN marker neuronal nitric oxide synthase (nNOS), while they formed synaptic connections (Magnusson et al., 2014). However, most newborn neurons generated in both studies have a short life-span and fail to express markers of fully differentiated striatal neurons, either due to their precocious death or lack of proper commitment, but attain complex and specific morphologies (Magnusson et al., 2014; Nato et al., 2015). Additionally, studies in early and late onset HD genetic models have revealed the presence of newborn neurons in the striatal area, that have either ectopically migrated from the SVZ (Kohl et al., 2010) or have originated from selective proliferation in the striatal area, respectively (Kandasamy et al., 2015). Importantly, intraventricular administration of BDNF/Noggin in R6/2 mice enhanced striatal neurogenesis and delayed motor impairment, implicating induced neurogenesis as an important contributor to functional recovery (Cho et al., 2007). Along the same lines, continuous administration of FGF2 in R6/2 mice not only stimulated SVZ neurogenesis and newborn neurons’ recruitment to the striatum, but also provided neuroprotection and prolonged survival of striatal neurons (Jin et al., 2005). In parallel, *in vivo* reprogramming has been a promising approach for the conversion of glial cells (astrocytes, NG2 glia and pericytes) into more mature, subtype-specific neurons under defined conditions using specific TFs both in the injured cortex (Heinrich et al., 2010, 2014; Guo et al., 2014) and normal striatum (Niu et al., 2013; Torper et al., 2013, 2015). In some of those studies the induced neurons were electrophysiologically functional and could integrate into the endogenous circuitry (Guo et al., 2014; Torper et al., 2015), highlighting the potential of this approach in producing functionally mature neurons *in vivo*. Recently the *in vivo* direct reprogramming approach has been successfully employed in a mouse model of Parkinson’s disease (Rivetti Di Val Cervo et al., 2017). In this study a combination of the TFs NEUROD1, ASCL1, and LMX1A with the microRNA miR-218 was used in order to reprogram adult striatal astrocytes into induced dopaminergic neurons that were shown to be excitable and managed to correct some aspects of motor behavior *in vivo*. However, it must be kept in mind that enhancement of endogenous neurogenesis, or *in vivo* reprogramming do not address the fact that mHTT is not targeted and is widely expressed throughout the brain, so a primary neurodegenerative process can occur in newly generated cells. However, as HD is an age related disease, a high rate of new neurogenesis may lead to tissue rejuvenation providing substantial benefit. Thus, *in vivo* direct reprogramming remains a promising approach for the production of healthy MSNs in the striatum with interesting and still largely unexplored potential, especially if the reprogramming process is combined with gene therapy strategies aiming at the down-regulation of mHTT in the reprogrammed newborn neurons. To this end, approaches to reduce mHTT levels *in vivo* in animal HD models are being tested over the last decade using either intrastriatal rAAV-mediated delivery of anti-mHTT shRNAs (Rodriguez-Lebron et al., 2005), antisense oligonucleotides (ASO) that catalyze RNase H-mediated degradation of mHTT mRNA (Kordasiewicz et al., 2012), or more recently miRNA-based mHTT mRNA-lowering using AAV viral vectors (Miniarikova et al., 2017). All these approaches so far (recently reviewed in Miniarikova et al., 2018) show promising results in alleviating HD symptomatology in animal models and could potentially be combined with regenerative therapeutic strategies and/or neuroprotective molecules in order to further enhance their therapeutic effect on HD progression. To this end, a recently released drug trial using the antisense oligonucleotide, IONIS-HTT_R_ that targets Huntingtin mRNA and suppresses mutant HTT production, has shown promise as a potential disease-modifying HD therapy (Tabrizi et al., 2018).

## The Role of miRnas in HD Therapeutic Approaches

As already mentioned, miRNAs (miR-9/9^∗^, miR-124, miR-218) have been already used in direct reprogramming protocols in combination with TFs to produce induced-striatal, cortical or motor neurons. Interestingly, besides their promising use in direct reprogramming protocols to slow down HD progression, miRNAs are also implicated in HD pathology and it has been very recently reported that disease-specific miRNAs have been detected in the cerebrospinal fluid (CSF) of HD patients (Reed et al., 2017) and could be potentially used as early HD prognostic markers. The perturbation of the neural miRNA system observed in HD, in many cases occurs through a mechanism involving the REST complex. REST complex targets include the neuronal specific microRNAs miR-9/9^∗^, miR-29a, miR-29b, miR-124 and miR-132 (Johnson and Buckley, 2009), all being dysregulated in human HD samples, or mouse models of HD. Conversely, components of the REST complex are targets for down-regulation by certain neurogenic miRNAs, such as miR-9/9^∗^ and miR-124, suggesting that their neurogenic potential is at least in part due to challenging REST complex levels and/or activity (Visvanathan et al., 2007; Packer et al., 2008), a concept that may have far reaching implications regarding experimental therapeutic strategies for HD. miR-124 is the most abundant miRNA in both the embryonic and adult CNS, acting globally to increase the expression levels of neuronal genes through several different pathways, including repression of anti-neural transcriptional repressor REST complex (Visvanathan et al., 2007). Dysregulation of miR-124 has been shown in many CNS disorders and conditions, including CNS tumors, inflammation and stroke (Sun et al., 2015). During HD progression in particular, the levels of miR-124, as well as other neurogenic miRNAs, are significantly reduced (Johnson and Buckley, 2009), resulting in disorganization of the neurogenic program. On the other hand miR-124 enhances striatal neurogenesis in HD, as striatal stereotaxic injection of miR-124 mimics increased striatal cells’ proliferation and improved motor function of R6/2 mice (Liu et al., 2015), while exosomal delivery of miR-124 in a mouse model of ischemia led to an increase in cortical neurogenesis and ameliorated the injury (Yang et al., 2017). However, the neurogenic/ neuroprotective potential of miR-124 in HD remains to be further studied, as in a recent study exosome-based delivery of miR-124 in the striatum of R6/2 mice did not lead to a behavioral improvement (Lee et al., 2017). In light of the evidence provided that the neural miRNA system is affected during HD progression, the mechanism of action of specific brain-enriched miRNAs in instructing or reinforcing neurogenesis or *in vivo* neuronal reprogramming would lead to the identification of new therapeutic strategies to combat HD.

## Neuroprotection as a Means to Reduce or Prevent Neuronal Degeneration in HD

It is obvious from recent studies that although significant advances have been made in the identification of molecular pathways and screening of potential drug targets using stem cells and reprogramming technologies (for review see (Connor, 2018), a stand-alone therapeutic strategy cannot reverse HD progression. Promoting neuronal replacement from endogenous sources and fostering neuroprotection of existing neurons are distinct but complementary strategies in view of devising an effective therapy in HD. Coupling these two approaches, ideally along with – as already mentioned – a strategy for the downregulation of mHTT, could have profound clinical impact. On the one hand induced-newborn neurons will rejuvenate the injured striatum, but require substantial support for their long-time survival and incorporation into the existing neural networks, while on the other hand the existing mature neurons need also support in order to alleviate their degeneration due to the wide presence of mHTT in the diseased brain. Thus, molecular mechanisms enhancing neurogenesis and neuroprotection represent valuable candidates in the field.

### Neuroprotective Protocols Aimed at Restoring BDNF Levels

Countless evidence shows that BDNF or BDNF/TRKB signaling is reduced in HD due to a mHTT-mediated mechanism. Produced by cortical neurons, BDNF promotes neuronal growth, survival of striatal neurons and plasticity. mHTT causes changes in vesicular transport of BDNF and transcriptional down-regulation of the BDNF gene. Indeed, BDNF levels are decreased in HD mouse models and in the brains of HD patients (Zuccato and Cattaneo, 2014). Restoration of BDNF levels is of interest in HD and delivery of BDNF through viral or stem-cell vehicles has shown some potential in inducing striatal neuronal regeneration, delaying motor impairment and extending survival in HD mouse models (Cho et al., 2007), but delivering a protein-based therapeutic to the CNS remains an important challenge. Because BDNF acts principally through binding to TRKB receptors, one approach is the developing of TRKB agonists and TRKB monoclonal antibodies. Several experimental compounds have been tested in HD mouse models and provided promising neuroprotective effects (Wild and Tabrizi, 2014). In particular small molecule modulation of p75NTR TRKB receptor has been shown to effectively reduce HD phenotype in mouse models providing evidence that targeting p75NTR may be an effective strategy for HD treatment (Simmons et al., 2013, 2016). Another innovative approach to restore BDNF levels is by inhibiting the formation of the REST-mSIN3 complex that is required for BDNF transcriptional repression. One compound has been identified, and encouraging results obtained in mHTT knock-in NSC lines (Conforti et al., 2013). Alternatively, as already discussed, neurogenic miRNAs and in particular miR-9/9^∗^ and miR-124 could serve as therapeutic agents to target the REST complex, as they have been both shown to down-regulate either REST itself (Packer et al., 2008) or other REST cofactors (Visvanathan et al., 2007). Thus, a combination of chemical compounds and miRNAs reducing REST complex formation may prove effective for HD treatment.

### Neuroprotective Approaches Acting on Metabolic Pathways

A big number of studies has highlighted mitochondrial defects leading to impaired energy metabolism and cellular oxidative stress in HD models and tissues of HD patients (Guedes-Dias et al., 2016; Liot et al., 2017). In this context, a potential target is the peroxisome proliferator-activated receptor (PPAR) gamma coactivator 1 alpha (PGC-1α), a transcriptional co-regulator of many nuclear-encoded mitochondrial genes. Expression of PGC-1α and its target genes get reduced in HD tissues and drugs able to activate PPAR nuclear receptors exert neuroprotective effects in both cellular and mouse HD models (Chiang et al., 2012). The ATPase valosin-containing protein (VCP), is another molecular target for HD as treatment with the HV-3 VCP deriving peptide that abolishes mHTT/VCP interaction corrects excessive mitophagy and reduces cell death in *in vitro* and *in vivo* HD models (Guo et al., 2016). Finally, a mitochondrial pathway which when targeted may provide neuroprotection in HD and other polyglutamine diseases is the activation of anti-apoptotic proteins belonging to B-cell lymphoma 2 (BCL2) family. As *in vitro* and *in vivo* models of HD and HD patients’ tissues show alterations in BCL2 family protein expression and localization (Sassone et al., 2013), it was suggested that inhibition of selected BCL2 family proteins may provide neuroprotection. Interestingly, BCL2 has been shown to act as a key factor for the improvement of glial-to-neuron direct reprogramming *in vitro* and *in vivo* by reducing cell death occurring due to the metabolic state transition of reprogrammed cells (Gascón et al., 2016).

Evidence also shows abnormal lipid metabolic pathways in HD and suggests that the development of new targets to restore their balance may act to ameliorate some HD symptoms (Desplats et al., 2007). A lipid pathway that dysfunctions in HD is the cholesterol metabolic pathway, as the expression of genes involved in the cholesterol biosynthetic pathway and the levels of cholesterol, lanosterol, lathosterol and 24S-hydroxycholesterol were found to be reduced in the brain of HD mouse models (Arenas et al., 2017). A recent study revealed a new cholesterol-targeting therapeutic strategy for HD through identifying abnormally low levels of the enzyme cholesterol 24-hydroxylase (CYP46A1) in HD models and in post-mortem brain tissues of HD patients. Delivery of CYP46A1 into the striatum of HD models decreased neuronal atrophy and improved motor deficits, implying that restoring CYP46A1 activity promises a new therapeutic approach in HD (Boussicault et al., 2016). Interestingly a nanoparticle-based cholesterol delivering strategy was able to restore synaptic and cognitive impairment in R6/2 HD mice, supporting the idea that therapies aimed to restore brain cholesterol level may have a significant impact in HD treatment (Valenza et al., 2015).

## Conclusion

Recent achievements in iPSC technology have contributed substantially to the understanding of the HD pathology and the screening for potential therapeutic molecules for HD. Furthermore, the advancements in *in vivo* neuronal reprogramming in different regions of the CNS, such as the cortex, the striatum, the spinal cord and the midbrain have opened new possibilities for the treatment of neurodegenerative diseases. Benefiting from those advances, a possible new approach for the treatment of HD could be a combination of promoting neuronal replacement from endogenous sources by direct reprogramming along with fostering neuroprotection by restoring BDNF levels or metabolic dysfunction of existing neurons, ideally in conjunction with the promising strategy of the down-regulation of mHTT levels.

## Author Contributions

JS and EP planned and wrote the manuscript. DT planned, wrote, and edited the manuscript.

## Conflict of Interest Statement

The authors declare that the research was conducted in the absence of any commercial or financial relationships that could be construed as a potential conflict of interest.
